# Truly continuous low pH viral inactivation for biopharmaceutical process integration

**DOI:** 10.1002/bit.27292

**Published:** 2020-02-24

**Authors:** Duarte L. Martins, Jure Sencar, Nikolaus Hammerschmidt, Andreas Flicker, Johanna Kindermann, Thomas R. Kreil, Alois Jungbauer

**Affiliations:** ^1^ Austria Centre for Industrial Biotechnology Vienna Austria; ^2^ Department of Biotechnology University of Natural Resources and Life Sciences Vienna Austria; ^3^ Department of Virology Global Pathogen Safety Takeda Vienna Austria

**Keywords:** virus inactivation, continuous processing, low pH viral inactivation, residence time distribution, virus clearance

## Abstract

Continuous virus inactivation (VI) has received little attention in the efforts to realize fully continuous biomanufacturing in the future. Implementation of continuous VI must assure a specific minimum incubation time, typically 60 min. To guarantee the minimum incubation time, we implemented a packed bed continuous viral inactivation reactor (CVIR) with narrow residence time distribution (RTD) for low pH incubation. We show that the RTD does not broaden significantly over a wide range of linear flow velocities—which highlights the flexibility and robustness of the design. Prolonged exposure to acidic pH has no impact on bed stability, assuring constant RTD throughout long term operation. The suitability of the packed bed CVIR for low pH inactivation is shown with two industry‐standard model viruses, that is xenotropic murine leukemia virus and pseudorabies virus. Controls at neutral pH showed no system‐induced VI. At low pH, significant VI is observed, even after only 15 min. Based on the low pH inactivation kinetics, the continuous process is equivalent to traditional batch operation. This study establishes a concept for continuous low pH inactivation and, together with previous reports, highlights the versatility of the packed bed reactor for continuous VI, regardless of the inactivation method.

## Graphical abstract

Virus inactivation is essential for product safety however its implementation in continuous mode remains a challenge while the biopharma industry aims at continuous biomanufacturing. This work describes the full viral inactivation of two model viruses by low pH using a packed‐bed reactor with narrow residence time distribution as incubation reactor.This novel approach enables an truly continuous operation while being as effective as the current batch approach.

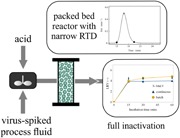

## 1 INTRODUCTION

Continuous viral inactivation (VI) is a key building block to complete the integrated continuous biomanufacturing process of the future (Johnson, Brown, Lute, & Brorson, 2017; Konstantinov & Cooney, 2015). The biopharmaceutical industry is following other industries in moving from discrete batch operation to integrated continuous manufacturing, especially for high demand products, such as monoclonal antibodies (MAbs; Shukla, Wolfe, Mostafa, & Norman, 2017; Walsh, 2018). The drivers for continuous processing are many‐fold; process intensification and cost savings (Baur, Angarita, Müller‐Späth, Steinebach, & Morbidelli, 2016; Hummel et al., 2018; Pagkaliwangan, Hummel, Gjoka, Bisschops, & Schofield, 2018; Pollock, Coffman, Ho, & Farid, 2017) might emerge as the most obvious ones but steady‐state operation and thus better, more reproducible quality have also been associated with continuous biomanufacturing (Karst et al., 2017; Kaufman, Wasley, & Dorner, 1988; Walther et al., 2019). While upstream processing is ahead in this transition, where chemostat and perfusion reactors are commonly employed at the manufacturing scale (Arathoon & Birch, 1986; Shukla et al., 2017; Warnock & Al‐Rubeai, 2006), downstream operations have only in recent years started this transition. Several purification steps have been developed or modified for continuous operation. The most pragmatic and simplest approach is to break down a unit operation into small sub‐processes and perform them in a cyclic or periodic manner (Gjoka, Gantier, & Schofield, 2017; Godawat et al., 2012; Jungbauer, 2013; PALL, 2018; Warikoo et al., 2012). However, with such an approach a discontinuous or discrete outflow is obtained, which renders process integration cumbersome or even impossible without numerous surge tanks. On the same line, parallelization of an entire sequence of multiple batch operations (protein A capture, low pH VI and polishing chromatography) has also been suggested (Flouquet & Banerjee, 2019) but with the same downsides and it requires a custom‐built skid for a specific process hindering facility flexibility. Truly continuous unit operations rely on novel systems and only a few examples are currently available (Casey, Gallos, Alekseev, Ayturk, & Pearl, 2011; Hammerschmidt, Sencar, Jungbauer, & Martins, 2019).

Low pH VI in batch mode is a simple process: the process intermediate is adjusted to the target pH, typically 3.5–3.7, incubated for 30–60 min and neutralized with high concentration buffer. In mAB processing, low pH VI is often integrated with protein A chromatography, since its elution is performed at a pH‐value close to those targeted for VI (Mattila et al., 2016). To avoid regulatory concerns about the possibility of hanging droplets or fluid inhomogeneities, a “two‐vessel” strategy might be employed. In short, the process intermediate is acidified in a first vessel, transferred to a second vessel and only then the incubation time starts (Shukla & Aranha, 2015). Continuous low pH VI must assure that critical process parameters (CPPs) stay within prevalidated operation limits throughout the whole process. Typically these include, at least, pH‐value, temperature, and minimum incubation time (Brorson et al., 2003; Mattila et al., 2016). Protein concentration and buffer salt might also impact the logarithmic reduction values (LRVs; Gillespie et al., 2018; Kreil & Roush, 2018). Temperature and pH‐value can be easily controlled through thermostats and in‐line mixers, respectively. Achieving the required minimum incubation is a more challenging feat, as a discrete incubation in batch translates into a residence time distribution (RTD). Recognizing the importance of RTD, the FDA draft guidance recommends RTD characterization for continuous processing (FDA, 2019). In strictly mathematical terms, the minimum incubation time for 100% of the fluid elements cannot be guaranteed given the statistical nature of the RTD determinations (also designated *E*‐curve). Alternatively, a minimum residence time (MRT) approach has been suggested, in which 99% or 99.5% of the fluid elements are incubated for at least the target incubation time, for example, 60 min (David et al., 2019; Klutz, Lobedann, Bramsiepe, & Schembecker, 2016; Martins et al., 2019). To satisfy the MRT approach, the time at which the cumulative RTD (also designated *F*‐curve) reaches, for example, 0.5% (*t*[*F*_0.5%_]) must equal 60 min—a typical minimum incubation time for low pH VI in batch mode.

For the specific case of continuous VI, the chance of any fluid element leaving the reactor before the target incubation time should be minimized, which highlights the requirement for a narrow RTD (Jungbauer, 2018). To achieve such a goal, three reactors have been patented and published. The coiled flow inverter (CFI) reactor (Klutz, Kurt, Lobedann, & Kockmann, 2015; Maiser, Schwan, Holtman, & Lobedann, 2016) and the jig‐in‐a‐box (JIB) reactor (Orozco et al., 2017; Coffman, Goby, Godfrey, Orozco, & Vogel, 2015) rely on Dean vortices to narrow the RTD in coiled open tube. Dean vortices—a secondary flow pattern that provides axial mixing—are generated by a fine balance of centripetal forces and centrifugal forces over a narrow Reynolds number range (Parker et al., 2018). An alternative approach based on the packed bed was suggested (Hammerschmidt et al., 2019). The bed of nonporous particles breaks the flow velocity profile thus resulting in a narrow RTD. Furthermore, the packed bed reactor outperforms the CFI and the JIB reactors of comparable scales in terms of RTD (Senčar, Hammerschmidt, Martins, & Jungbauer, 2020). Other systems have been suggested for continuous VI, however, some are not truly continuous in nature but rather cyclic (Gjoka et al., 2017; PALL, 2018) or do not consider the RTD when designing the incubation time (Arnold, Lee, Rucker‐Pezzini, & Lee, 2019; Vaidya et al., 2018; Xenopoulos, 2013, 2015).

The metric chosen for RTD narrowness measurement is also of great importance. Given the fact that peak fronting (i.e., early exit) is the greatest concern in a continuous VI process, the initial peak steepness is preferred for reactor assessment over other metrics that rely on the fitting of the whole *F*‐curve, such as the Bodenstein number (Senčar et al., 2020). The initial peak steepness is the ratio of the time at which the cumulative RTD reaches 50% and a defined threshold percentage, for instance, 0.5% (*t*[*F*_50%_]/*t*[*F*_0.5%_]) and has become the preferred characterization method/metric for a reactor's performance (Klutz et al., 2016; Martins et al., 2019; Orozco et al., 2017). The choice of the threshold percentage must be small enough to describe peak fronting and early‐existing fluid elements, but must be also large enough to be reproducibly monitored by commonly used detectors available for column performance testing and tracers generally regarded as safe (Amarikwa, Orozco, Brown, & Coffman, 2018). Overincubation should also be considered, especially for continuous VI designed according to the MRT approach where, by definition, most of the fluid elements are incubated longer for longer than the equivalent batch time. It is thus clear that the RTD analysis should also consider peak tailing (e.g., *t*[*F*_0.5%_]/*t*[*F*_99.5%_] has been used before [Klutz et al., 2015]). Overincubation might lead to product loss or damage, for instance, mAB aggregation at low pH (Joshi, Shivach, Kumar, Yadav, & Rathore, 2014). However, the published data for continuous VI systems do not suggest strong tailing in the RTD (Klutz et al., 2015; Orozco et al., 2017; Senčar et al., 2020) and such evaluation is the process‐ and product‐specific (Liu et al., 2016). One common concern in continuous processing is performance deterioration, namely changes in CPPs, with consequences for product quality and safety. Bed stability and bead shrinkage/swelling should be considered, especially at acidic pH‐values and if reactor components are known to be affected by environmental pH (Andersson Trojer, Wendel, Holmberg, & Nydén, 2012).

Besides assuring the CPP and robustness throughout the continuous operation, it is crucial to demonstrate low pH viral inactivation with relevant model viruses. Regulatory guidelines recommend the measurement of the VI kinetics described as “a biphasic curve in which a rapid initial phase is followed by a slower phase” (Committee for Proprietary Medicinal Products, 1996; International Conference on Harmonization, 1999). The same guidelines suggest different viruses that can be used based on cell‐line susceptibility, historic record, and relevance. In 2018, a publication assessed viral clearance using continuous low pH VI using a straight tube reactor (Gillespie et al., 2018) but unfortunately, no quantitative measurement of the RTD (ideally, initial peak steepness) was provided. More recently, a report detailed the continuous low pH VI using the CFI reactor (David et al., 2019). Besides the continuous inactivation, the report also covers the integration with the preceding unit operation (protein A continuous chromatography), which was achieved with a homogenization loop. Aside from low pH inactivation, continuous solvent/detergent (S/D) treatment using a packed bed continuous viral inactivation reactor (CVIR) has been shown by our group (Martins et al., 2019).

In the present work, we extend the concept of the narrow RTD, packed bed CVIR and describe its application for low pH treatment. The CVIR is characterized and the impact of MRT based on the RTD is discussed. Analysis of bead and bed stability under acidic conditions is provided. Two industry‐relevant model viruses are used (a) to control for any equipment‐induced viral inactivation and (b) to assess the effectiveness of the CVIR for continuous low pH viral inactivation and show process performance equivalent to batch mode.

## 2 MATERIALS AND METHODS

### 2.1 Reagents

All chemicals were of analytical grade and were purchased from Merck Millipore (Germany), unless otherwise stated.

### 2.2 Test item, test item buffer, and acid stock

The test item represents a generic process intermediate from a biopharmaceutical process. The test item consisted of 130 mM glycine (pH 7.0 ± 0.1), 8.84 ± 0.88 g/L human serum albumin. The test item buffer consisted of 130 mM glycine (pH 7.0 ± 0.1). A solution of 2 M glycine (pH 2.7) was used as acid stock to achieve the target pH‐value (pH 3.7 ± 0.1).

### 2.3 Viruses and cells

The pseudorabies virus (PRV), Strain Kaplan (kindly provided by Dr. Rhiza, Eberhard Karls Universität Tübingen, Germany), was propagated and titrated on Vero cells (ECACC 84113001) as described before (Unger, Poelsler, Modrof, & Kreil, 2009). Xenotropic murine leukemia virus (X‐MuLV) was propagated on Mus dunni cells (ATCC CRL‐2017) and titrated on PG4 cells (ATCC CRL‐2032) as described before (Martins et al., 2019).

#### 2.3.1 Virus infectivity assay

Infectious virus titers were determined by the median tissue culture infective dose (TCID_50_) assay, using eightfold replicates of 12 serial half‐log sample dilutions of virus‐containing samples that were titrated on the cell lines indicated above. For this, 100 µl per sample were added per well of the 96‐well plate, each seeded with 100 µl cell suspension. The cells were incubated at 36°C for 7 days before the cytopathic effect was evaluated by visual inspection under an inverted microscope (Nikon Eclipse TS100; Nikon, Japan). The TCID_50_ titers were calculated according to the Poisson distribution and expressed as log_10_(TCID_50_ ml^−1^). Virus reduction factors were calculated in accordance with the EU Committee for Proprietary Medicinal Products guidance (Committee for Proprietary Medicinal Products, 1996).

### 2.4 CVIR characterization and controls

**Figure 1:**
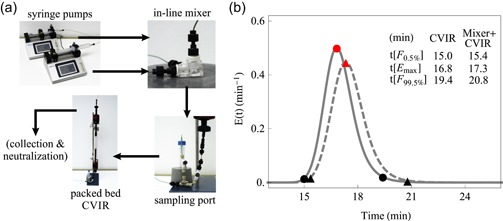
System setup depicting the key components. (a) The continuous VI setup is comprised of two syringe pumps used to drive both streams, an in‐line mixer for homogenization, a sampling port placed before the reactor and the packed bed CVIR. The arrows denote the fluid stream. The probability density function for the RTD of CVIR (full line) and in‐line mixer connected before the CVIR (dashed line) at 19.6 cm/hr. (b) The circles and triangles highlight *F*_0.5%_, *E*_max_ and *F*_99.5%_ for the CVIR alone and for the in‐line mixer connected before the CVIR, respectively, and their corresponding time is given in the top right insert. *F*_0.5%_ is used to define the CVIR volume accordingly with the MRT approach. CVIR, continuous viral inactivation reactor; RTD, residence time distribution; VI, viral inactivation [Color figure can be viewed at https://wileyonlinelibrary.com]

The continuous VI system is depicted in Figure 1a and consists of two syringe pumps used to drive both the test item and the acid stock. Both streams were continuously mixed in a custom‐built in‐line mixing chamber optimized for low void volume (0.48 ml). While other technologies are available for continuous in‐line mixing, such as static mixers, these would result in significant precolumn volume thereby biasing the total exposure time for small‐scale setups. The usage of the custom‐built in‐line mixer enables more efficient mixing due to the power input while having a limited contribution to the system precolumn volume. The precolumn volume, namely the in‐line acidification mixer volume, should be as low as possible to (a) avoid increasing the exposure to inactivating conditions for substantially longer times than that established by the MRT approach (especially important for validation studies and comparison against batch) and (b) minimize RTD tailing of the whole system (as shown in Figure 1b). Note the limited contribution of the mixer to the overall RTD and, more important, to RTD tailing (the *t*[*F*_0.5%_]/*t*[*F*_99.5%_] ratio decreases from 0.773 to 0.739 due to the mixer). For the CVIR, 300 µm poly(methyl methacrylate) (PMMA) beads (PLPM‐300; Kisker Biotech GmbH, Germany) were packed in a HiScale 16 column (GE Healthcare Life Sciences, Sweden). A custom‐built vibration cage was used to aid packing. The packed CVIR had a height of 137 mm, an internal diameter of 16 mm and a void volume of 11.1 ml (which represents 40.3% of the geometric volume, 27.5 ml).

#### 2.4.1 CVIR characterization

Frontal analysis with a noninteracting tracer (2% [vol/vol] acetone in water) was performed to characterize the CVIR. The cumulative RTD (*F*‐curve) was registered and the initial peak steepness (*t*[*F*_0.5_]/*t*[*F*_0.005_], specific for continuous VI) as well as traditional chromatography metrics (height equivalent to a theoretical plate [HETP], in µm, and asymmetry at 10% peak height) were calculated. The HETP was calculated based on the moments method and was corrected for the system contribution in bypass without column. The linear velocity investigated ranged from 5 to 300 cm/hr (or from 0.167 to 10.1 ml/min, respectively). The asymmetry was calculated based on the derivative of the acetone front.

In this study, we designed and operated the reactor according to the MRT approach to guarantee that 99.5% of the fluid elements are incubated for the respective target time. More precisely, the definition of the reactor volume (*V*_R_) relevant for continuous VI equals the volume at which the cumulative RTD (*F*‐curve) reaches 0.5% (*V*[*F*_0.5%_]), meaning *V*_R_ =* V*[*F*_0.5%_] = 9.85 ml at 19.6 cm/hr (or 0.657 ml/min, the highest linear velocity/flow rate employed for VI studies and hence the worst case for fronting). Note that the (cumulative) RTD is typically shown as a function of time but can be shown as a function of volume by conversion with the volumetric flow rate. Throughout this study, the incubation times (*t*) in a continuous mode referred are calculated based on the volumetric flow rate, *Q*_total_, and the *V*_R_ according to *t* =  *V*_R_/*Q*_total_—this reflects the ever‐present objective of assuring at least the respective incubation time. However, using the MRT approach also means that the majority of the fluid elements are incubated longer than the design criterion (*t*[*F*_0.5%_]). For instance, in the case of the 15‐min incubation experiments (defined by *t*[*F*_0.5%_] = 15 min), the modal incubation time (*t*[*E*_max_]) is 17 min—11.3% longer than the design criterion (Figure 1b). This slight overincubation is a feature common to all continuous VI processes designed based on the MRT approach.

#### 2.4.2 CVIR stability under acidic pH

##### Bead swelling/shrinkage

PMMA particles in different conditions (dry, in aqueous suspension at neutral pH and in aqueous suspension at pH 3.0) were analyzed in a phase‐contrast microscope (Olympus CKX53SF with ×4 objective). The images were recorded using an Olympus SC50 digital image acquisition module (Olympus Europa, Germany). The beads were deposited on a six‐well plate and analyzed on‐plate under the different conditions to avoid sampling artifacts. For pH 3.0 incubation, a 50 mM sodium citrate, 150 mM NaCl (pH 3.0) buffer was used. The choice of pH 3.0 was selected as a worst‐case scenario. Image processing (component analysis) was performed in Mathematica 11 (Wolfram) to identify circle‐like shapes and their respective diameter was calculated. Each image/analysis was visually validated and images with more than one misidentified particle were excluded from statistical analysis.

##### Packed bed stability

A 130‐mm HiScale 16 column was packed with 300‐µm diameter PMMA beads and frontal analysis (with 1 M NaCl) was performed to determine initial peak steepness. Then, the CVIR was exposed for up to 10 days to 50 mM sodium citrate, 150 mM NaCl (pH 3.0) buffer. For this experiment, NaCl was chosen to avoid changes in concentration due to evaporation. The initial peak steepness was measured at different points during the exposure period. The frontal analysis was performed five times per time point and the linear velocity was 300 cm/hr to represent a worst‐case scenario.

### 2.5 Viral inactivation method

#### 2.5.1 Generation of pH titration curve

To establish the acid stock addition rate a pH titration was performed with spiked test item (see Section 2.5.2). The acid stock was added under stirring and the pH‐value was monitored.

#### 2.5.2 CVIR‐induced virus loss control

A spiked test item and the test item buffer (130 mM glycine [pH 7.0 ± 0.1]) were used to mimic the viral inactivation experiment at neutral pH. The CVIR setup was operated at *Q*_total_ of 0.657 and 0.164 ml/min (or 4.90 and 19.6 cm/hr, corresponding to 15‐ and 60‐min incubation, respectively, based on *F*_0.5%_) with both model viruses. The *Q*_total_ is the total flow rate through the CVIR or the addition of the spiked test item and acid stock flow rates. Samples for virus titration were collected from the spiked test item, from the mixer outlet stream, from the CVIR outlet stream after 1.0, 2.0, 3.0, 4.0, and 5.0 *V*_R_ of operation and from the hold control (HC).

#### 2.5.3 Batch low pH viral inactivation

For batch VI, a process intermediate (affinity chromatography eluate) was used as a test item. After temperature adjustment to 16 ± 1°C, the test item was filtered through a 0.45‐µm syringe filter (SLHV033RS; Merck Millipore, Germany). Because of the eluate's acidic pH, part of the test item was neutralized with 1 M Tris (pH 8.5) and then spiked with virus stock to provide samples for spike control (SC), that is initial titer, and for HC. The SC was immediately titrated and the HC was only titrated after the low pH batch VI process was completed. The remainder of the test item was spiked and immediately adjusted to pH 3.7 ± 0.1 with 2 M glycine buffer (pH 2.7). The pH‐adjusted, spiked test item was incubated under continuous stirring at 16 ± 1°C for 59 ± 1 min. Samples for TCID_50_ virus titration were drawn 15 ± 1, 30 ± 1, and 60 ± 1 min after pH adjustment and immediately neutralized by 10‐fold dilution with PBS.

#### 2.5.4 Continuous low pH viral inactivation

The CVIR setup was buffered with the test item buffer. The spiked test item and the acid stock were loaded into 50 and 5 ml syringes, respectively. To achieve the incubation time of 15, 30, and 60 min according to the MRT approach, the volumetric flow rate, *Q*_total_, was 0.657, 0.328, and 0.164 ml/min (or 19.6, 9.80, and 4.90 cm/hr), respectively. The custom‐built mixer was primed and a sample for pH‐value confirmation was drawn. The CVIR was operated for at least 5 *V*_R_, after which an HC sample was drawn for comparison against the spiked test item sample. A sample was collected at the outlet of the CVIR after 1.0, 2.0, 3.0, 4.0, and 5.0 *V*_R_ of operation and 0.2 ml were immediately transferred and diluted 10‐fold with PBS for pH neutralization. All continuous experiments were performed at 16 ± 1°C inside a temperature‐controlled laminar flow hood. The temperature at the CVIR wall was measured and logged with a Pt‐100 probe and µR1000 recorder (Yokogawa America). The CVIR setup was sanitized between experiments with 0.5 N NaOH for 10 min.

## 3 RESULTS AND DISCUSSION

The CVIR was first characterized with respect to its RTD—this is the first step to implement a continuous VI process. The packed bed CVIR was additionally assessed regarding its stability towards prolonged low pH exposure, both in terms of particle size and packed bed RTD. The ratio between spiked test item and acid stock flow rates was established and the pH‐value at the CVIR's inlet and outlet were confirmed. Control experiments were implemented under non‐inactivating conditions to show that the CVIR system does not contribute to VI. Extensive VI data with two model viruses demonstrated comparability of LRV values to batch data.

### 3.1 System controls

#### 3.1.1 CVIR characterization

**Figure 2:**
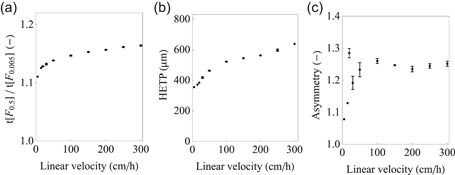
(a) Column characterization by the *t*[*F*_0.5_]/*t*[*F*_0.005_] metric, (b) height equivalent to a theoretical plate (HETP) in µm, and (c) asymmetry at 10% peak height

The VI reactor is based on the packed bed principle to achieve a narrow RTD. Traditionally, packed bed packing quality relevant for bioseparation chromatography is typically assessed by the HETP and asymmetry. In addition, and more importantly, the initial peak steepness—*t*[*F*_0.5_]/*t*[*F*_0.005_], the preferred metric to characterize continuous VI reactors (Senčar et al., 2020)—is provided. The CVIR characterization (Figure 2) shows a general improvement of all three metrics with linear velocity reduction. The initial peak steepness reached 1.11 at 4.90 cm/hr (or 0.164 ml/min and equivalent to 60‐min incubation in 137‐mm high, 16‐mm diameter column). The initial peak steepness is close to that of an ideal case, that is *t*[*F*_0.5_]/*t*[*F*_0.005_] = 1 for a perfect plug flow, which highlights narrow RTD/good packing and is comparable other reports (Senčar et al., 2020). Increasing the linear velocity to up to 300 cm/hr (or 10.1 ml) has a limited impact on initial peak steepness (see Figure 2a), which highlights the flexibility and simplicity of operation of the packed bed CVIR. Such flexibility—the possibility to operate at either 1 or 60 min incubation time with narrow RTD—has not been demonstrated for reactors based on Dean vortices, possibly because narrow RTD is achieved for a limited range of Reynolds number, Dean number, and coil‐to tube diameter (Klutz et al., 2015; Parker et al., 2018; Rossi, Gargiulo, Valitov, Gavriilidis, & Mazzei, 2017). The HETP increased with increasing linear—this is expected as hydrodynamic dispersion dominates in this range of linear velocities (Carta & Jungbauer, 2010; the linear velocity range assessed—4.90–300 cm/hr—is equivalent to a reduced velocity range of 3.58–219 in the van Deemter plot). At the lowest linear velocity, the peak is very close to symmetric, however, as the linear velocity increases, the asymmetry increases to 1.2–1.3, denoting peak tailing. One could speculate that tubing, the mesh and frit or other system components can act as a mixing chamber and contribute to tailing (remember that the column void volume is only 11.1 ml and even a small mixing spot would induce tailing, characteristic in the RTD of a continuous stirred tank reactor). It is important to highlight, that HETP and asymmetry (or even porosity) provide the only context for traditional bioseparation chromatography. For continuous VI, packing quality and reactor performance should be evaluated by a metric especially sensitive to peak fronting (or “early exit”). Initial peak steepness, *t*[*F*_0.5_]/*t*[*F*_0.005_], has been used to characterized continuous VI reactors by independent groups (Klutz et al., 2016; Martins et al., 2019; Parker et al., 2018). Furthermore, initial peak steepness is especially designed for increased sensitivity to fronting and outperforms other metrics based on the whole RTD curve, such as the Bodenstein number (Senčar et al., 2020).

#### 3.1.2 Particle stability at low pH

**Figure 3:**
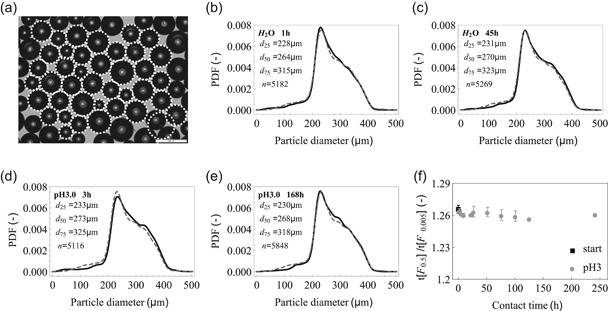
Poly(methyl methacrylate) (PMMA) stability at acidic pH. Exemplary results of particle identification and determination by image processing. (a) The dotted circles denote the particle identified by the image processing tool. The scale bar is 500 µm. PMMA particle diameter probability density function (PDF) upon exposure to different conditions. (b–e) The gray dashed line represents the reference data (dry beads) and the solid black line represents the respective condition. The quartiles of the diameter distribution and the number of successfully identified particles are shown in the top, left inset. (f) PMMA packed bed stability upon exposure to pH 3.0. The initial peak steepness was measured five times per contact time point and the average ± standard deviation is shown

Considering the final intended application, the stability of the PMMA particles as well as of the packed bed itself was evaluated at pH 3.0 (Figure 3). To assess PMMA bead swelling or shrinkage, multiple microscope fields were analyzed by an image processing script. The result of a single analysis is shown in Figure 3a, where the software‐identified particles are shown to match the actual particles. To maximize the number of observations per condition, a compromise of one misidentified particle per field was accepte;, images with more than one misidentified particle were excluded from the analysis. Incubation of the particles in water for up to 45 hr (Figure 3b,c) did not change the particle size distribution when compared with the dry state. Similar results were obtained when the PMMA beads were incubated in citrate pH 3.0 for up to 7 days (Figure 3d,e). Also, only negligible changes in the particle diameter quartiles (*d*_25_, *d*_50_, and *d*_75_) were found across different conditions. The initial peak steepness did not change considerably upon exposure to low pH (Figure 3f).

The PMMA stability data indicates that both the loose beads and the packed bed are unaffected by extended exposure to pH 3.0 as observed by others (Albeladi, Al‐Romaizan, & Hussein, 2017; Ali, Bt. Abd Karim, & Buang, 2015). A constant *t*[*F*_0.5%_], and more generally a narrow RTD, is critical throughout the operation as it relates directly with VI time—a CPP. Based on the stability shown at pH 3.0, it is expected that the PMMA beads and the packed bed remain unchanged at pH‐values typically used in VI, for example, pH 3.5–3.9. Despite showing bead and bed stability, the time frame was limited and shorter than campaign duration typically envisaged for continuous processes, that is 30 or 60 days. Implementation in such a process would have to assess the PMMA stability over the required campaign duration, after which it would be disposed of.

#### 3.1.3 Achieving the target pH

**Figure 4:**
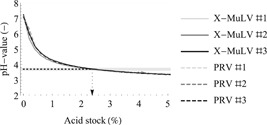
Titration to low pH. The test item spiked with either model virus (X‐MuLV in full line or PRV in dashed line) was titrated with the acid stock (2 M glycine [pH 2.7]) to determine the flow rate ratio of both streams for continuous VI runs. For each virus, the titration was performed in triplicate. The dotted line and arrow show the pH target (pH 3.7) and the respective acid fraction. The gray highlight shows the acceptable pH range (pH‐target ± 0.1). PRV, pseudorabies virus; VI, viral inactivation; X‐MuLV, xenotropic murine leukemia virus

While in batch mode, the pH can be easily adjusted by the addition of concentrated acid and simultaneous pH measurement, in the continuous mode, this approach is not feasible. The simplest way to overcome this limitation is to preliminarily titrate the spiked test item with the acid stock to determine the volume of acid stock needed to reach the target pH (Figure 4). An acid volumetric fraction of 2.38% is needed to acidify the test item to pH 3.7 ± 0.1, independently of the virus stock used. Titration of the spiked test item is dependent on the test matrix and acid stock used, and therefore it must be performed on a case‐by‐case basis (Gillespie et al., 2018) when the pH feedback control loop is not available (typically the case for small‐scale process development and validation).

**Figure 5:**
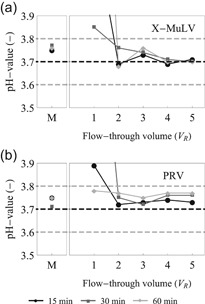
pH‐value measurement throughout the continuous operation for (a) xenotropic murine leukemia virus (X‐MuLV) and (b) pseudorabies virus (PRV) experiments. Samples were drawn for off‐line pH confirmation after the mixer (M) and at the reactor outlet after 1, 2, 3, 4, and 5 *V*_R_ of operation for 15, 30, and 60 min continuous incubation (black circles, dark gray squares, and light gray diamonds, respectively). The solid lines link the adjacent points and serve only as visual aids. The dashed lines represent the pH target and range (pH 3.7 ± 0.1)

The pH‐value was confirmed both at the reactor inlet and at the reactor outlet throughout the continuous operation (Figure 5). The pH‐value is a CPP in any low pH VI process, hence tight monitoring and control are required. As expected, the in‐line mixer was effective in homogenizing the spiked test item and the acid stock to reach pH 3.7 ± 0.1. After the initial ramp‐up phase, which lasts until 2 *V*_R_, the pH‐value at the reactor outlet was constant and within the targeted range (pH 3.7 ± 0.1). The stable pH from 2 *V*_R_ onwards is an indication that the system reached steady‐state operation. Based on the inlet and outlet pH‐values, it is fair to assume that the pH inside the column is within the target pH range. The use of inert, non‐functionalized PMMA beads also supports this assumption, as there is no ion exchange between the fluid phase and the stationary phase inside the packed bed CVIR.

#### 3.1.4 CVIR‐system controls

**Figure 6:**
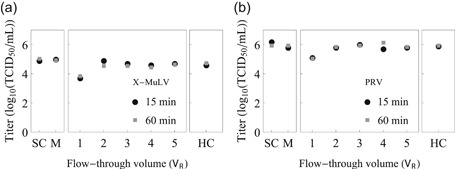
Control experiments at neutral pH with (a) xenotropic murine leukemia virus (X‐MuLV) and (b) pseudorabies virus (PRV). For each model virus, the extreme flow rates were tested, which correspond to 15 and 60 min incubation (black circles and gray squares, respectively). The spiked test item control (SC), the mixer's outlet (M), the continuous reactor's outlet at 1, 2, 3, 4, and 5 *V*_R_ and the hold control (HC) were sampled for virus titration

To exclude any possible impact of our system on virus infectivity a series of controls under noninactivating conditions was performed. Control experiments are recommended in industry guidelines (International Conference on Harmonization, 1999) and are likely to be especially relevant due to the novelty of the employed system as well as due to the shear‐labile nature of virus particles (Wolff & Reichl, 2011). The control experiments (Figure 6) show that the titers at the mixer's outlet and CVIR's outlet are similar to those of the starting material (4.95 and 6.15 log_10_(TCID_50_/ml) for X‐MuLV and PRV, respectively). However, there is a notable exception: the samples at 1 *V*_R_ for both incubation times and for both model viruses. This is explained by the fact that 1 *V*_R_ lies in the process ramp‐up phase, in accordance with the reactor volume definition by the MRT approach. Based on the reactor volume definition (*V*_R_ =* V*[*F*_0.5%_]), at 1 *V*_R_, the expected titer is 0.5% of the initial titer. However, the titers registered are 1.0–1.4 log_10_(TCID_50_/ml) above the expected titer. In practice, an infinitely small sample is not the case, but rather the sample covers a volume before and after 1 *V*_R_ (or *V*[*F*_0.5%_]), so a titer above that based on the RTD is expected. Additionally, a contributing factor is the sampling process, in which a sample is drawn at the CVIR outlet (~0.5 ml) and only part of if (0.2 ml) is diluted for neutralization and titrated, thus the excess of sampled material can contribute to the higher titer. It is noteworthy, that the influence of the sampling process is only relevant for the ramp‐up phase, as in steady‐state the fluid elements before and after have the same composition and incubation time, leading to the same VI performance. That is the case of the samples at 2 *V*_R_ and onwards, which show a constant titer, suggesting steady‐state operation (as supported also by the RTD characterization, Figure 1b, which indicates that steady state is achieved after 1.3 *V*_R_, i.e., *F*_99.5%_). The lack of system‐induced VI is especially important for, and simplifies, process validation as any possible system‐induced VI would have to be controlled for and the underlying phenomenon would have to be clarified (International Conference on Harmonization, 1999). The lack of system contribution means that the entirety of the VI can be attributed to the respective mode of action, in this case, exposure to low pH.

### 3.2 Continuous VI and comparison against batch

**Figure 7:**
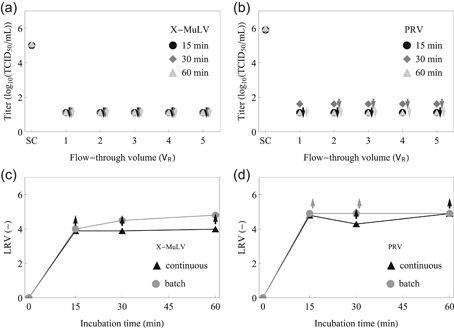
Continuous low pH viral inactivation. Viral titer for (a) X‐MuLV and (b) PRV throughout the continuous experiments. The SC denotes the starting concentration of the spiked test item. The flow‐through volume from 1 to 5 *V*_R_ denotes the operation duration. The incubation inside the reactor was 15, 30 or 60 min (black circle, dark gray diamond, and light gray triangle, respectively). The downward arrow denotes a sample below the TCID_50_ limit of detection. Viral inactivation kinetics at low pH for (c) X‐MuLV and (d) PRV. The average ± standard deviation LRV registered by continuous operation (black triangles) is compared against equivalent data generated in batch mode (gray circles). Note that, due to the constant titers, the standard deviation is 0, which renders the error bars invisible. The upward arrow denotes minimum LRV due to the respective sample being below the limit of detection. The lines link the adjacent points and serve only as visual aids. LRV, logarithmic reduction value; PRV, pseudorabies virus; SC, spike control; TCID_50_, median tissue culture infective dose; X‐MuLV, xenotropic murine leukemia virus

The VI runs were performed at different flow rates to achieve 15, 30, and 60 min incubation (Figure 7a,b). For the model virus X‐MuLV, the low pH viral inactivation was fast, resulting in no detectable infectivity even for the shortest incubation time tested (15 min). Full inactivation of X‐MuLV was also registered after 14.5 min exposure to low pH using the CFI reactor (David et al., 2019). In the case of the PRV, extensive inactivation was also found in most of the samples drawn throughout the operation. Because the viral titer was already below (or at) the TCID_50_ limit of detection, longer incubation times (30 and 60 min) did not result in further titer reduction. Based on the initial and final viral titer, the average LRVs were calculated (Figure 7c,d; note that no error bars are provided because the final titers were constant between 2 and 5 *V*_R_, thus resulting in the same LRV). For the reasons described above, the sample at 1 *V*_R_ was excluded from the LRV calculation, since it corresponds to the ramp‐up phase. The LRV enables the assessment of the VI effectiveness as well as a comparison against the corresponding batch process. In fact, the VI kinetics obtained from the continuous operation is similar to that obtained in batch, which suggests comparable effectiveness of both operation modes. This observation is in agreement with a different study recently published, where a packed bed CVIR was implemented for continuous S/D inactivation (Martins et al., 2019). These results indicate comparability of the packed bed CVIR to the traditional batch operation—a likely regulatory concern.

## 4 CONCLUSION

A truly continuous low pH viral inactivation process was implemented using a narrow RTD‐packed bed reactor. The same CVIR was characterized at linear velocities ranging from 4.90 to 300 cm/hr, corresponding to incubation times from 1 to 60 min. It is important to highlight the packed bed system's flexibility with respect to incubation time, since increasing the linear velocity had limited negative impact on the initial peak steepness and RTD, which is not the case for Dean vortex‐based reactors, like the JIB (Brown, Orozco, & Coffman, 2019). It was demonstrated that the PMMA beads withstand exposure to pH 3.0 for multiple days. Additionally, the low pH had no adverse impact on the packed bed, which means that the incubation time—a CPP—is maintained throughout the operation. Despite the PMMA stability demonstrated, implementation in longer continuous biomanufacturing campaigns (e.g., 60 days) requires additional studies. Monitoring of the pH‐value demonstrated that steady state is achieved within only 2 *V*_R_, after which the pH and the VI capacity was constant. In the case of 60‐min continuous VI, the ramp‐up phase would last no more than 2 hr, which represents a small fraction (0.28%) of a hypothetical 30‐day continuous campaign. The RTD can provide additional information about the ramp‐up phase but it should not be used to establish steady‐state. Defining the steady state based only on the RTD has the problem of which cumulative RTD threshold should be used. Is steady‐state reached at *F*_99.5_%? or at *F*_99.99_%? The answer is not trivial, especially given the logarithmic nature of virus titers/clearance. For this reason, we argue that steady‐state operation must be supported by data showing constant CPPs (e.g., pH‐value for continuous VI) and constant performance (e.g., LRV for continuous VI). Therefore, the pH inside the reactor was not adjusted to pH 3.7 ± 0.1 before starting the experiment. For manufacturing purposes, the pH inside CVIR can and should be preadjusted to the target pH‐value and the decision on whether to discard or further process the ramp‐up effluent would be subject to process integration considerations (e.g., can the subsequent unit operation process a stream with increasing protein concentration). Control experiments at neutral pH showed no system‐induced VI. On the other hand, low pH continuous incubation resulted in extensive VI for both industry‐relevant model viruses. Even at 15 min continuous incubation, an LRV of ≥3.9 and ≥4.8 was registered for X‐MuLV and PRV, respectively. Furthermore, the low pH VI kinetics in continuous mode was shown to be as effective as the batch operation for low pH VI (as it was shown before for the case of S/D treatment [Martins et al., 2019]). This technology represents a viable option to complete a fully integrated, truly continuous process for acid‐tolerant biopharmaceuticals, such as mABs.

## 5 OUTLOOK

The integration of continuous VI into a continuous process is a challenging task. Most protein A periodic counter‐current chromatography (PCC) reports describe the use of three or four columns (Pollock et al., 2013; Warikoo et al., 2012; Zydney, 2016), which leads to a periodic and discontinuous outflow from the unit operation. To cope with a discontinuous mass flow, a surge tank might be necessary to accommodate the elution from a PCC system before continuous VI. More complex implementations of continuous chromatography are possible (Steinebach, Müller‐Späth, & Morbidelli, 2016) and with increased column numbers; for example, 12 columns (David et al., 2019), a continuous outflow might be realized, thus simplifying integration. Independently of whether an undisrupted mass flow is assured, homogenization of stream from the preceding unit operation (e.g., continuous protein A elution) and acidification to the target pH is needed. Such objectives can be combined into one single stage for an undisrupted mass flow scenario (David et al., 2019) or combined with a surge tank in a case of interrupted mass flow. Homogenization is not exclusively linked to pH‐value and a stable protein concentration (possibly a CPP) is desirable. Constant protein concentration and consequentially viscosity are especially important for reactors, whose narrow RTD is enabled by Dean vortices, as such secondary flow patterns depend heavily on the solution's viscosity and its acceptable range must be studied (Brown et al., 2019). The unit operation subsequent to the continuous VI should be taken into consideration as well. Can the subsequent unit operation cope with varying product concentrations observed before the continuous VI? The mixer and packed bed (or any other narrow RTD) reactor will not dampen such fluctuations significantly. If the following unit operation is not affected by changes in product concentration and other potential CPPs, a continuous VI might be realized without surge tanks, providing that undisrupted mass flow is assured. If the following unit operation requires a constant‐composition stream, then one surge tank is required either before or after the continuous VI. The size of the surge tank and concomitant RTD broadening should be the topic of careful consideration, as the large vessels would dampen fluctuations to a greater extent but would also propagate an out‐of‐specification case to a larger volume with its potential loss. Studies on the whole process's RTD are necessary and will be of invaluable help in such decisions.

The backpressure generated by one individual unit operation is also a topic of concern when integrating multiple unit operations. For instance, in the case of JIB, a pressure drop below 5 psi was cited as a design goal (Orozco et al., 2017). Contrarily to traditional chromatography, where pressures exceeding 5 psi are common, the packed bed CVIR is characterized by low back pressure due to (a) the larger particles used (200–400 µm in diameter) and (b) the lower linear velocities employed for continuous VI (e.g., 4.90–19.6 cm/hr). As an illustrative example, the Carman–Kozeny equation predicts a back pressure of 0.0586 kPa (or 0.00850 psi) at 19.6 cm/hr, which makes a reliable measurement impossible with the standard pressure monitors in typical chromatography workstations (Senčar et al., 2020).

Validation of a continuous VI is also a topic of concern. Besides inactivation studies with live viruses (inactivation kinetics), demonstration of stable process parameters might be also required. While parameters like pH‐value or temperature can be easily measured, measuring the RTD throughout the operation is not easily performed. In a continuous VI processes designed according to the MRT approach, the RTD (or at least *V*[*F*_0.5%_]) could be viewed as a CPP. How this question is addressed depends on the system used to provide continuous incubation. Recently, Brown et al. (2019) published a data‐driven approach to characterize the impact of viscosity on the Dean number/RTD narrowness and provide an empirical relationship for the JIB scale up. Our group has characterized extensively the packed bed reactor and how a feature like a particle size, linear velocity, or packed bed geometry contribute to the initial peak steepness (*t*[*F*_0.5_]/*t*[*F*_0.005_]) (Senčar et al., 2020). Despite the data produced, it is likely that RTD measurement of the actual reactor employed in manufacturing would still be required as a performance test both before and after use (similarly to an integrity test for a viral filter). Such an approach implies that a GMP‐compliant tracer must be used, thus limiting how low the threshold in the MRT approach can be.

## CONFLICT OF INTERESTS

The authors declare that there are no conflict of interests.
